# EFFECTS OF OBESITY ON EXECUTIVE FUNCTION AND MEMORY IN MIDDLE-AGED AND OLDER ADULTS

**DOI:** 10.1093/geroni/igz038.2418

**Published:** 2019-11-08

**Authors:** Andrew J Fiscella, Ross Andel

**Affiliations:** University of South Florida, Tampa, Florida, United States

## Abstract

Obesity has been linked to multiple conditions including cardiovascular disease, cancer, and metabolic syndrome. While the relationship between obesity and these diseases is well-established, the relationship between obesity and changes in cognitive function is less clear. This study examined the effects of overweight and obese status on performance in tests of executive function and memory. It was hypothesized that overweight and obese individuals would perform worse than normal weight individuals. Data from 547 individuals who participated in the Midlife in the United States Refresher cohort were used. Body mass index (BMI) and waist circumference (WC) were employed as measures of obesity. Both variables were examined continuously and categorically (from NHBLI-defined cutoffs). Z-scores were computed for each cognitive test and averaged together to create separate composite scores for executive function and memory. When measuring BMI and WC continuously, higher BMI was significantly associated with lower executive function (B=-0.010, p=.029); no significant association between executive function and WC was found (p=.162). When measuring BMI and WC with clinical thresholds, participants with overweight or obese WC had significantly lower executive function (p=.030). No overall association between BMI-based thresholds and executive function was found (p=.682). Memory did not differ between overweight/obese participants and healthy weight controls with continuous or clinical threshold specifications. These findings suggest that: 1) obesity may relate to poorer executive function but not worse memory, and 2) the way in which obesity is measured reflects saliently in the results. Future researchers should consider conducting sensitivity analyses with different obesity criteria.

